# What Factors Encourage Young People to Engage in Substance Use? Substance Use and Associated Factors Among Youth in Southwest Ethiopia: A Community-Based Study

**DOI:** 10.3389/fpubh.2022.796687

**Published:** 2022-03-14

**Authors:** Tinsae Abeya Geleta, Berhanu Senbeta Deriba, Dejene Edosa Dirirsa

**Affiliations:** ^1^Department of Public Health, College of Health Sciences, Salale University, Fitche, Ethiopia; ^2^Department of Midwifery, College of Health Sciences, Salale University, Fitche, Ethiopia

**Keywords:** substance use, substance and alcohol use, khat (Catha edulis), alcohol drinking, cigarette smoking

## Abstract

**Background:**

Substance use indicated the use of psychoactive substances such as alcohol, cigarettes, khat, and illegal drugs. Substance use has varying impacts on the health and socio-economics of countries, and is a major public health concern globally. Currently, substance use is a common public health problem among Ethiopian youth mainly in the city of Jimma. Therefore, this study aimed to assess the magnitude of Cigarette smoking, alcohol drinking, khat chewing, and associated factors among the youth of Jimma town in 2019.

**Methods:**

A community-based cross-sectional study was conducted among youth of Jimma town from March 2019 to April 2019. A simple random sampling technique was used to select 423 study participants. Data were collected using a structured interviewer-administered questionnaire. The collected data were entered into EPI data manager version 4.4.1 and transported to SPSS version 23 for data cleaning and analyses. The disruptive study was carried out to determine the prevalence of cigarette smoking, alcohol consumption, and khat chewing. Binary and multivariable analyses were carried out to identify factories associated with cigarette smoking, alcohol consumption, and khat chewing. Finally, adjusted odds ratios (AOR) with 95% confidence intervals (CI) were used to determine the presence and strength of association.

**Results:**

The current prevalence of cigarette use, alcohol use, and khat use was 16.0, 30.6, and 45.7%, respectively. Factors associated with current smoking use were substance use by siblings, subjective norm factors, and perceived benefits of substance use. Factors associated with current alcohol consumption were youth who highly perceived substance use as important. Factors associated with current khat use were male, substance use by siblings, out-of-school youth, and subjective norms.

**Concussion:**

The study findings indicated that the prevalence of khat, alcohol, and cigarettes was high among the youth of the city of Jimma. To reduce the prevalence of khat, alcohol, and cigarettes among youth, coordinated efforts from the youth, the government, health professionals, and the community at large are needed.

## Background

Substance use is termed the use of psychoactive substances such as cigarettes, khat, alcohol, and illegal drugs (1). Psychoactive substance use can lead to dependence on behavioral, cognitive, and physiological phenomena that develop after repeated substance use (1). Substance use is a comprehensive term that includes the taking of all substances within which there are stages such as substance-free, that is, non-user, experimental user, recreational user, and harmful user (2).

The surveys conducted on substance use among general worldwide populations show that the extent of substance use among young people remains higher than among older people, with some exceptions associated with the traditional use of drugs such as opium or khat (3). According to the literature, early (12–14 years old) to late (15–17 years old) adolescence is a critical risk period for substance use initiation, and substance use is highest among young people aged 18–25 years (4). According to a meta-analysis conducted in Ethiopia, the most commonly used substances in Ethiopia were khat, cigarettes, alcohol, and shisha (5).

According to the WHO global report on trends in the prevalence of tobacco use, among young people aged 15–24 globally, the prevalence rate of tobacco use has declined from 22.6% in the year 2000 to 17.0% in 2015. The prevalence rate in 2025 is projected to be 14.2% (6). Rendering to the 2018 WHO global alcohol status report, the prevalence of alcohol consumption among youth aged between 15-19 and 20–25 was 45.7 and 48.5, respectively (7).

The Ethiopian Demographic and Health Survey (EDHS) conducted in 2016 showed that women in the age group between15–19 and 20–24 who drank alcohol are 30.4 and 34.1% respectively. Male in the age group of 15–19 and 20–24 who drank alcohol is 39.1 and 46.4% respectively. It also reports that women in the age group 15–19 and 20–24 have ever chewed khat, 7.4%, and 10.0%, respectively. Male in the same age group who ever chewed chat are 13.8 and 23.8 respectively. Women in the age group between 20-24 who smoke tobacco are 1.0 % and men in the age group between 15–19 and 20–24 who smoking tobacco are 0.4 and 2.6 respectively (8).

Substance use and dependence are the most frequently occurring disorders in the young and general population. A significant proportion of the youth population uses substances and it has adversely affected their health, school performance, and interpersonal relationships (2). Additionally, substance use decreased academic performance, increased the risk of exposure to HIV/AIDS and other sexually transmitted diseases (STDs), and also causes psychiatric disorders (2).

This study was initiated on the basis of the following rationale. First, substance-related problems were not adequately addressed, and the problem is now growing alarmingly in our country. Furthermore, the Ethiopian minister of health identified it as a problem among young people and adolescents, and the Jimma zone health bureau identified it as the primary problem among young people, alongside HIV/AIDS. This study also attempted to address a new dimension in that it was a community-based study conducted among a more vulnerable group of youth aged 15 to 25. In Ethiopia, only a few articles on the substance's use on youth at the community level have been published. The finding of this study is useful for counseling and providing health education on the effect of substance use. Additionally, identifying factors associated with substance use among youth at the community level is essential to guide program planning, support youth to adhere to protective issues, and it is also important for the community to increase circumstances for the application of preventive factors and to decrease persuading factors, finally serving as input for policymakers.

## Methods

### Study Design, Period, and Setting

A community-based cross-sectional study was used from March 2019 to April 2019. The study was carried out in Jimma Town. Jimma town is located in Jimma Zone, Oromia Regional, State, and southwest Ethiopia. Jimma was the capital city of the Jimma Zone, and it is found 345 km away from Addis Ababa, the capital city of Ethiopia. The Town has 17 Kebeles with a total population of 205,163 of whom women account for 102,007, male, 103,156, and a total household of 42,742 according to the 2019 Jimma zone health bureau projection population report. Of the total population, around 40,539 (21%) populations are youth (age found 15–24).

### Population

All youth (15–25 years) who lived in the town of Jimma were the source population. All randomly selected youth who lived in the selected Kebeles were considered the study population. All youth (15–25 years old), who lived in Jimma city for more than 6 months and who were present at the house during the data collection period, were included in the study. Young people who were unable to respond due to a severe illness and were not willing to participate in the study were excluded.

### Sample Size Determination and Techniques

**Figure 1:**
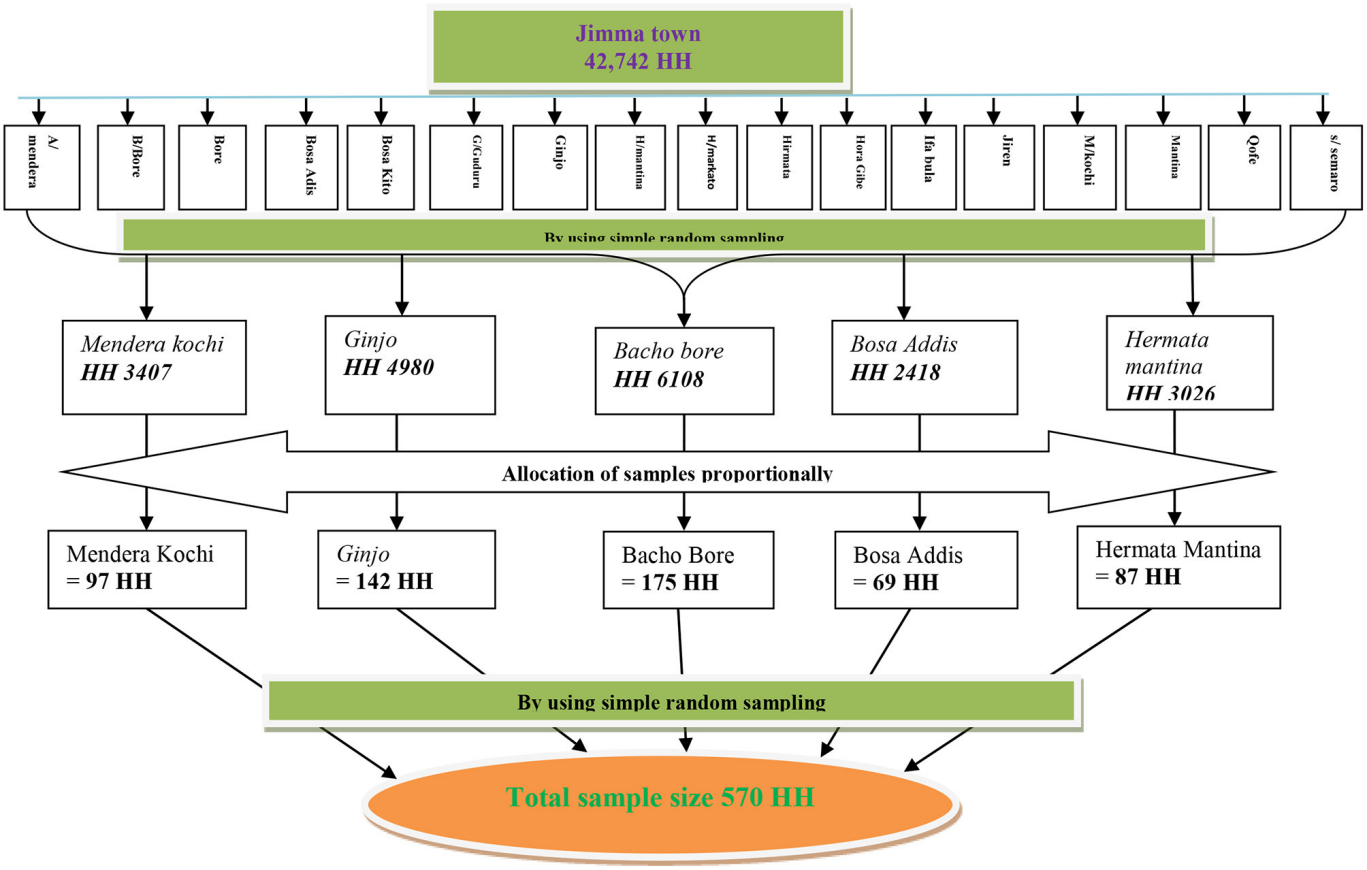
Schematic representations of sampling techniques.

The sample size was calculated using a single population proportion formula taking the prevalence rate of khat users from the previous study (9). With a 95% confidence level, 5% precision, and a non-response rate of 10 %. The total sample size is 345 ^*^ 1.5 design effect ^*^ 10% nonresponse rate = **570**. A multistage sampling technique was used to recruit study participants. Jimma town was selected purposefully by taking into account the magnitude of substance use (e.g., Khat) in this specific area. Simple random sampling methods were used to select five (30%) out of 17 Kebeles in the Jimma town, namely; Mendera-Kochi, Bacho Bore, Matina Markato, Ginjo Guduru, and Bosa Addis. The number of the household in each Kebele was obtained from the Jimma zone health bureau. The sample size was then assigned in proportion of households in each selected Kebeles (Figure 1). Finally, households were selected using a simple random sampling technique specifically computer-generated random number was used to obtain the required sample size. Household lists were taken from the selected kebele health post office. Their usual place of residence was identified in collaboration with Kebele leaders. For more than one youth present in the households, one youth was selected by using the lottery method, and also for no youth in the household the house was jumped to the next house.

### Data Collection Instruments and Procedure

Data were collected using a structured questionnaire. The data collection tool is adapted after reviewing different kinds of literature (9–13). The data were collected through an interviewer-administered questionnaire on the weekends to include both in-school and out-of-school students. If the youth were not present due to class makeup or other issues during data collection, the data collectors returned to the house at least three times to contact youth who were not present during the first attempt. The data collection questionnaire was developed in English and translated into the locally spoken language (Afan Oromo and Amharic), and then back-translated into English by language experts to check its consistency. The questionnaire was contented with six sections 1, sociodemographic, 2, cigarettes use, alcohol use, Khat use assessing questions 3, factors related to social life 4, subjective norm 5, psychological factors, and 6, factors related to substances. Five health professionals (five diplomas) and one supervisor (one bachelor's degree) participated in data collection. To ensure the quality of data, a pre-test was done before the actual data collection period. About 28 youth (5%) of the sample size households were used for this purpose from nonselected Kebeles (Seto Semero), and some points found on the questionnaire are improved. Before starting a real data collection, supervisors and data collectors were trained for one day about the study's objective, purpose, the ethical issue of the study, and data collection technique.

### Study Variables

#### Dependent Variables

Current khat use, current alcohol use, and current cigarette use.

#### Independent Variables

Individual-related factor, socio-demographic related factor, substance-related factor, psychological factor, and social-related factor.

### Operational and Term Definition

**Substance**: The commonly used substances, such as alcohol, cigarettes, and khat, induce changes in thinking, feelings, and behavior that can cause dependence. **Substance Use:** substance uses referred that taking alcohol, cigarettes and khat to alter mood or behavior. **Current khat user**: a person who chewed chat at least once in the past 30 days. **Current alcohol user**: a person who drink alcohol at least once in the past 30 days. **Current cigarette user**: a person who smoking cigarette at least once in the past 30 days. **Subjective norm**: Substance use favoring of the community is measured by a Likert question, which contains three questions (Most people who are important to me will approve of my substance usage khat, cigarette, alcohol), Most people who are important to me think that, I should use substance (khat, cigarette, alcohol) and Most people who like me want my engaging in substance use (khat, cigarette, alcohol) and a computed score of three items, indicating the maximum sum score considered as important to the respondent's approved substance use (13). **Psychological factor**: The effect of psychological problems (stress, mental distress, and depression) on substance use is measured by the Likert scale, which contains four questions (I use substances (khat, cigarette, alcohol) to relieve tension, I use substances (khat, cigarette, alcohol) to relieve depression, I use substances (khat, cigarette, alcohol) to Feel relaxed and I use substances (khat, cigarette, alcohol) to Forget my problem), with the computed sum score of four items indicating the maximum sum score considered as a psychological factor. **Perceived benefit of substance use**: considering substances as useful things is measured by the Likert scale, which contains three questions, with the computed sum score of three items indicating the maximum sum score considered as a high benefit of substance use as perceived by the user (13). **Substance-related factors:** includes substance availability, accessibility, and affordability it is measured by a Likert scale that contains six questions, and the computed score of six items of the Likert scale indicating the maximum sum score is considered as high Substance-related factors.

### Data Analysis

The data collected were entered into Epi data manager version 4.4.1 and transferred to SPSS version 21.0 for data analysis purposes. After the data was cleaned for the missing value, the data were analyzed using descriptive and inferential statistics. Specifically, frequency and percentages were used for descriptive analysis, and bivariate and multivariate logistic regression analyzes were used to identify factories associated with cigarette smoking, alcohol consumption, and khat chewing. The degree of dependence (current use of khat, current use of alcohol, and current use of cigarettes) and the independent variable association was assessed using an odds ratio, and statistically significant were declared at 95% of confidence intervals (CI) and the *p*-value (Pv) less than 0.05. The goodness of the test model was checked by the Hosmer-Lemeshow goodness fit, and the *p*-value of the model fitness test was 0.28, 0.31, and 0.42 for cigarette use, alcohol use, and khat use, respectively.

## Results

### Sociodemographic Characteristics of the Respondents

A total of 567 youth from Jimma town participated in the study, with a response rate of 99.47%. Of the study participants, 397 (70%) are male and 170 (30%) are female. Youth between the ages of 18–20 constitute about 34.9% of respondents (Table 1).

**Table 1:** Sociodemographic characteristic of the study participants Jimma, south-west Ethiopia, 2019.

**Variable**	**Category**	**Frequency**	**Percent (%)**
Sex			
	Male	397	70.0
	Female	170	30.0
	Total	567	100
Age	15–17	184	32.5
	18–20	198	34.9
	21–24	185	32.6
	Total	567	100
Current	In school	321	56.6
youth status	Out of school	246	43.4
	Total	567	100
Level of	Illiterate	71	12.5
education	Grade 1–8	210	37.0
	Grade 9–10	156	27.5
	Grade 11–12	65	11.5
	Diploma and above	65	11.5
	Total	567	100.0
Religion	Muslim	309	54.5
	Orthodox	156	27.5
	Protestant	92	16.2
	Other	10	1.8
	Total	567	100.0
Frequency	Never	24	4.2
of visiting	Few times a year	13	2.3
worshiping	Once a month	18	3.2
	Every week	298	52.6
	Every day	214	37.7
	Total	567	100.0
Living	With family	327	57.7
arrangement	With relatives	82	14.5
	With friends	114	20.1
	Alone	44	7.7
	Total	567	100.0

### Prevalence of Cigarettes

Ninety-one (16.0%) study participants currently smoked a cigarette and among these, 47.1% of the respondents started smoking cigarettes between the ages of 16–20 years. Most, 86 (84.3%) reported that their friends introduced them to smoking cigarettes. Forty-three (42.2%) of the respondents argued that they smoke a cigarette because it made them happier. Seventeen-two (70%) of the respondents were smoking 1–5 sticks of cigarette per day, 81 (79.4%) smoke a cigarette at Khat house, and 538 (94.9%) of the study participant knows the effect of smoking a cigarette on human health (Table 2).

**Table 2:** Prevalence of cigarettes among youth in Jimma town, southwest Ethiopia, 2019.

**Variable**	**Category**	**Frequency**	**Percent (%)**
Ever smoked a cigarette in the last 30 days?			
	Yes	91	16.0
	No	10	1.8
Age at first smoked cigarette			
	10–15	47	46.1
	16–20	48	47.1
	21–24	7	6.8
	Total	102	100.0
Introduce to use of cigarettes			
	Friend	86	84.3
	Parents	4	3.9
	Relatives	1	1.0
	Out of curiosity	11	10.8
	Total	102	100.0
Convinced into smoke Cigarette			
	Make one brilliant	30	29.4
	Happier	43	42.2
	Stronger/healthier	3	2.9
	Have confidence	13	12.8
	Boost appetite	13	12.7
	Total	102	100.0
How many cigarettes smoked per day			
	1–5 sticks	72	70.6
	6–10 sticks	7	6.9
	1 packet	23	22.5
	Total	102	100.0
Where do you smoke the cigarette?			
	At home	12	11.8
	Bar	5	4.9
	At khat chewing	85	83.3
	Total	102	100.0

### Factors Associated With Current Smoking in Multivariate Logistic Regression

The multivariate logistic regression model identified that the use of sibling substances, the subjective norm factor, and the perceived benefits of substances was significantly associated with current cigarette smoking with a *p* < 0.05. Respondents whose siblings use substances were identified to be 2.5 times more likely to use cigarettes than those siblings who do not use the substance (AOR 95%CI 2.53 [1.31-4.87)]. In this study, youth who were influenced by the subjective norm factors were 15% more likely to smoke a cigarette [AOR 95%CI 1.15 (1.02-1.30)]. Young people who perceived substance use as important were 42% more likely to smoke a cigarette [AOR 95%CI 1.417(1.16-1.73)] (Table 3).

**Table 3:** Results of the bivariate and multivariate logistic regression analysis showing the factor associated with cigarette smoking among the youth of Jimma, southwest Ethiopia, 2019.

**Variable**	**Category**	**Ever smoking a cigarette in the last 30 days**		**COR 95%CI**	***P*-value**	**AOR 95% CI**	***P*-value**
		**No**	**Yes**				
Sibling substance use							
	Yes	120(21.2%)	59(10.4%)	5.439 (3.374–8.768)	0.001	2.529 (1.313–4.871	0.006
	No	354(62.7%)	32(5.7%)	1			
Perceived benefit	3.45 (±SD) 1.8			2.021 (1.730–2.361)	0.001	1.417 (1.160–1.730)	0.001
Subjective norm	4.21 (±SD) 2.24			1.439 (1.318–1.572)	0.001	1.153 (1.023–1.300)	0.02

### Prevalence of Alcohol

Of the total of participants, 170 (30.0%) reported that they have currently drunk alcohol. Among these, 88 (49.4%) start drinking alcohol at the age of 10–15, and 122 (68.5%) frequently use beer/drink. Of the youth who drank alcohol, 118 (66.3%) reported that they did so under peer pressure and 118 (66.3%) were drinking alcohol to make themselves happier (Table 4).

**Table 4:** Prevalence of alcohol among youth in Jimma town, southwest Ethiopia, 2019.

**Variable**	**Category**	**Frequency**	**Percent (%)**
Ever drunk alcohol in the last 30 days			
	Yes	170	30.0%
	No	8	1.4
Age first had drunk alcohol			
	10–15	88	49.4
	16–20	84	47.2
	21–24	6	3.4
	Total	178	100.0
Frequently drink alcohol			
	Beer/draft	122	68.5
	Wine	8	4.5
	Sprit	8	4.5
	Whisky	1	0.6
	Local drinks	34	19.1
	Mixed drinks	5	2.8
	Total	178	100.0
Who introduces you to using alcohol?			
	Friend	118	66.3
	Parents	40	22.5
	Relative	7	3.9
	Out of curiosity	10	5.6
	Sibling	3	1.7
	Total	178	100.0
Convinced into the drink			
	Happier	118	66.3
	Stronger/healthier	21	11.8
	Work long hours	7	3.9
	Have confidence	16	9.0
	Boost appetite	16	9.0
	Total	178	100.0
Ever used alcohol and other substances			
	Yes	87	48.9
	No	91	51.1
	Total	178	100.0
What substance that you use at the same time			
	Khat	74	85.1
	Cigarette	13	14.9
	Total	87	100.0
Drinking alcohol causes serious illness			
	Yes	536	94.5
	No	31	5.5
	Total	567	100.0
Where do you drink alcohol?			
	At home	63	35.4
	Friend place	64	35.9
	In bar	40	22.5
	Relatives place	11	6.2
	Total	178	100.0

### Factors Associated With the Current Alcohol Drinker in Multivariate Logistic Regression

The multivariate logistic regression model identified that the perceived benefit of substance use was significantly associated with current alcohol consumption with a *p* < 0.05. Young people who perceived substance use as important were 72% more likely to drink alcohol [AOR 95% CI 1.715 (1.5-2.03)] (Table 5).

**Table 5:** Bivariate and multivariate logistic regression analysis showing the factor associated with alcohol consumption among the youth of Jimma town, southwest Ethiopia, 2019.

**Variable**	**Category**	**Ever drunk alcohol in the last 30 days**		**COR 95%CI**	***P*-value**	**AOR 95% CI**	***P*-value**
		**No**	**Yes**				
Perceived benefit	3.45 (±SD) 1.8			1.784 (1.598–1.992)	0.001	1.715 (1.448–2.032)	0.001
Types of substance used by father	Cigarette	9 (3.3%)	11 (4.1%)	3.013 (1.186–7.655)	0.020	2.543 (1.090–6.982)	0.005
	Alcohol	20 (7.4%)	30 (11.1%)	3.698 (1.945–7.034)	0.001	2.624 (2.651–11.932)	0.001
	Khat	143 (52.8%)	58 (21.4%)	1		1	

### Prevalence of Khat

The study revealed that about half 259 (45.7%) of the study respondents have currently chewed khat. Amongst these, 140 (52.6%) reported that they start chewing khat at the age of 10–15 years. The majority of 207 (77.8%) of the study participants started chewing khat due to peer pressure. Of the youth who ever chewed khat, 124 (46.6%) used it occasionally and 97 (36.5%) chewed khat for relaxation and entertainment (Table 6).

**Table 6:** Prevalence of khat among youth in Jimma town, southwest Ethiopia, 2019.

**Variable**	**Category**	**Frequency**	**Percent (%)**
Ever chewed khat in the last 30 days			
	Yes	259	45.7
	No	7	2.6
Age at first starting chewing Khat			
	10–15	140	52.6
	16–20	119	44.7
	21–24	7	2.7
	Total	266	100.0
Who introduces you first to using chat?			
	Friend	207	77.8
	Parents	29	10.9
	Relative	5	1.9
	Out of curiosity	25	9.4
	Total	266	100.0
How often do you use khat			
	Occasionally	124	46.6
	Monthly	46	17.3
	Weekly	31	11.7
	Daily	65	24.4
	Total	266	100.0
Why do you use khat			
	Increase in concentration	85	32.0
	Stronger/ work hard	35	13.1
	Because my friends chew	47	17.7
	Relaxation and entertainment	99	37.2
	Total	266	100.0
Ever use khat and other substances at the same time			
	Yes	111	41.7
	No	155	58.3
	Total	266	100.0
If yes, what substances use at the same time			
	Alcohol	69	62.2
	Cigarette	42	37.8
	Total	111	100.0
Does chewing khat cause serious illness?			
	Yes	483	85.2
	No	69	12.2
	I don't know	15	2.6
	Total	567	100.0

### Factors Associated With Current Khat Chewing in Multivariate Logistic Regression

The multivariate logistic regression model revealed that sex, substance use by siblings, current school status, subjective norm factor, and psychological factor were significantly associated with the current chewing of khat at *p* <0.05. In this study, male respondents were eight times more likely to chew khat than female respondents [AOR 95%CI 8.33 (4.24–16.36)]. Respondents whose siblings use substances were 3.6 times more likely to chew khat than those whose siblings do not use the substance [AOR 95%CI 3.76(2.15-6.57)]. Out–of–school youth were 3.36 times more likely to chew khat than those who were in school [AOR 95%CI 3.36(1.99-5.64)]. In this study, youth who were highly influenced by subjective norms were 22% more likely to chew khat [AOR 95%CI 1.22(1.06-1.39)]. Young people with a psychological problem were 52% more likely to chew khat [AOR 95%CI 1.52(1.39-1.66)] (Table 7).

**Table 7:** Bivariate and multivariate logistic regression analysis showing the factor associated with khat chewing among the youth of Jimma town, southwest Ethiopia, 2019.

**Variable**	**Category**	**Ever chewing khat in the last 30 days**		**COR 95%CI**	***P*-value**	**AOR 95% CI**	***P*-value**
		**No**	**Yes**				
Sex							
	Male	162 (28.6%)	235 (41.4%)	8.825 (5.483–14.202)	0.001	8.33 (4.242–16.361)	0.001
	Female	146 (25.7%)	24 (4.2%)	1			
Currently school status							
	In school	218 (38.4%)	103 (18.2%)	1			
	Out school	90 (15.9%)	156 (27.5%)	3.669 (2.586–5.204)	0.001	3.356 (1.996–5.643)	0.001
Sibling substance use							
	Yes	42 (7.4%)	137 (24.2%)	7.144 (4.755–10.734)	0.001	3.758 (2.149–6.570)	**0.001**
	No	265 (46.9%)	121 (21.4%)	1			
Subjective norm	4.21 (±SD) 2.24			1.641 (1.469–1.833)	0.001	1.215 (1.056–1.397)	0.006
Psychological factors	6.83 ±SD 3.47			1.611 (1.499–1.732)	0.001	1.518 (1.390–1.658)	0.001

## Discussion

This study assessed the prevalence of cigarette smoking, alcohol consumption, and khat chewing and associated factors among youth from Jimma town, Oromia, Ethiopia. This study finding revealed that 91 (16.0%) study participants currently used cigarette and sibling substances, subjective norm factor, and perceived benefit of substances were the main factor associated with cigarette smoking. One hundred and seventy (30.0%) of the study respondents were current consume alcohol and the perceived benefit of substance was the factor associated with current alcohol consumption. The study revealed that about half 259 (45.7%) of the study respondents were current chewed khat and sex, use of sibling substances, current school status, subjective norm factors, and psychological factors were significantly associated with current chewing. of khat.

The current prevalence of smoking is 91 (16.0%), slightly higher than the study accompanied in Jimma town 10.2% (14), Bale preparatory school students 5.6% (12), Bonga public college student 1.5% (15), Woreda high school adolescents 6.8% (11), Axum university 9.3%, Ethiopian demographic and health survey report 4.4% (8), Nigerian secondary school student 4.7% (16) and the survey conducted in Sudan 13.7 % (17). This discrepancy occurred due to a difference in study population and study design. The current study was conducted on the youth of Jimma town (including both in-school and out-of-school youth) and also conducted through community-based. The previous study was conducted on in-school youth, and most of the in-school youth waste their time on education when compared with out-of-school youth. In the current study, around 43% of the study participants were out-of-school youth. The high rate of cigarette smoking was linked to easy access and availability of cigarettes, as well as the lack of a strong smoking control rule.

The current prevalence of alcohol is 170 (30.0%). This finding is in line with the study conducted on a Mekele university student (18) and it is slightly higher than the study accompanied Bale preparatory school students 23.6% (12), Debre Berhan university student 16.9% (19), Nigerian secondary school 8.9% and conducted in Sudan 2.7 % (16). This discrepancy occurred due to a difference in study population and study design. The current study was conducted on the youth of Jimma town (included both in-school and out-of-school youth) through a community based cross sectional study. The previous study was conducted on university and high school youth. Most of the time, university and high school students were busy due to their educational load when compared to out-of-school youth. In the current study, around half of the participants were out-of-school youth, and they had a lot of free time to drink alcohol. In contrast, this finding was lower than the study conducted on Bonga public college students 44.9 (15) and Woreta high school adolescents 40.9% (11). Low alcohol use might be associated with a high number of Islamic religious followers. In the current study, about 55% of the study participants were Islamic religious followers. The Islamic community considered drinking alcohol as a sin. Therefore, alcohol drinking was not accepted by the community.

The current prevalence of chewing khat is 259 (45.7%). These findings are higher than the study conducted on Jimma university internship medical student 13.4% (20), Jimma town high school students 14.2% (14), Bonga public college student 11.4% (15), Woreta high school adolescents 13.8%, Bale preparatory school students 5.6% (12), Debre Berhan university student 5.7% (19) and Ethiopian Demographic and Health Survey report (8). The current study finding was higher than previous study conducted in different parts of Ethiopia due to difference study population, design and setting. The rational justification for this finding is that khat is readily available, accessible, and affordable in the study area due to the local community's economic reliance on khat tree cultivation and some of the Islamic religious followers' use it for praying, or “duway”, but the religion does not command this practice. Also, the community considered khat chewing as normal.

In this study, we identified that the respondents whose siblings use substances were 2.5 times more likely to use cigarettes than those siblings who do not use a substance. This finding is consistent with the previous study (21–26). This finding could be explained by the fact that young people tend to mimic and exercise what they see their family members do. In this study, the subjective norm factor was significantly associated with current smoking. This study finding is in line with a study conducted on Woreta high school adolescents (11) and Nepal Rithepani high school adolescents (27). And also a study conducted on high school students in north Italy showing that seeing teachers who smoke a cigarette was significantly associated with cigarette smoking (24). This might result from exposure to such behaviors through social and mass media and also due to the government police's weakness in substance use controls.

Our finding indicated that the perceived benefit of substance use was significantly associated with alcohol drinking. Most youths in Ethiopia drink alcohol for relaxation and entertainment purposes and are also considered depressant substances after chewing khat or taking other substances. The study revealed that being male was significantly associated with chewing khat. This finding is in line with the previous study conducted in Ethiopia (14, 28–31) and also a study conducted in Saudi Arabia (31). It can be argued that the community more accepts substance use among males more easily than females. In this study, the use of the substance by siblings was significantly associated with the current chewing of khat. This finding is in line with previously conducted studies (30, 32, 33). This finding could be explained by the fact that young people tend to mimic and exercise what they see their family members do.

The youth found out of school was more likely to chew khat than those youth found in school. The possible justification for this finding was an academic failure, lack of a job, and lack of a recreation area were the main factors that motivated youth to use substances. This study revealed that the psychological factor is significantly associated with chewing khat. This finding was consistent with the study conducted on Gonder university students (33). This is due to the strong link between psychological problems and substance use. But the study conducted in Jimma town contradicts this finding, khat chewers are ten times more likely to develop depression than non-chewers (34).

The subjective norm factor of the community was significantly associated with the chewing of khat by youth. This finding is in line with a study conducted on Woreta high school adolescent students (11). The possible justification for this finding is that the community norms favorable to substance use encouraged young people to engage in substance use. The limitation of the study was that most of the discussion part was compared with the study conducted on high school and university students due to a limited number of articles on youth at the community level.

## Conclusions And Recommendation

Generally, this study finding indicated that the prevalence of khat, alcohol, and cigarettes was high among the youth of the town of Jimma; specifically, the prevalence of khat was highest among the youth of the Jimma town than the other two substances. Factors associated with smoking were siblings' smoking, subjective norm factors, and perceived benefits of substance users. Alcohol consumption was significantly associated with the perceived benefits of substance users. Khat chewing was significantly associated with sex; siblings' khat chewing of siblings, current youth, school status, subjective norm factors, psychological factors. Therefore, to alleviate these factors, coordinated effort from youth, the government, and the community at large is required. Public health professionals, specifically health promotion and health behavior experts, should focus on providing health education to youth in order to reduce the prevalence of substance use, by providing detailed education on the consequences of substance use on one country's health, social life, and economy. and developing appropriate information education and communication and behavioral change communication materials. Family members or siblings must provide a good example by abstaining from using substances.

## Data Availability Statement

The raw data supporting the conclusions of this article will be made available by the authors, without undue reservation.

## Ethics Statement

The studies involving human participants were reviewed and approved by Jimma University Ethical Review Board. Written informed consent to participate in this study was provided by the participants' legal guardian/next of kin.

## Author Contributions

TG, DD, and BD were involved in the writing of the manuscript, interpretation of the results, and performed the statistical analysis. TG was involved in the data collection process and study coordination. All authors read and approved the final manuscript.

## Conflict of Interest

The authors declare that the research was conducted in the absence of any commercial or financial relationships that could be construed as a potential of interest.

## Publisher's Note

All claims expressed in this article are solely those of the authors and do not necessarily represent those of their affiliated organizations, or those of the publisher, the editors and the reviewers. Any product that may be evaluated in this article, or claim that may be made by its manufacturer, is not guaranteed or endorsed by the publisher.

## Abbreviations

AOR: Adjusted Odd Ratio
COR: Crude Odd Ratio
CI: Confidence interval
EDHS: Ethiopian Demographic and Health Survey
HIV: Human Immune Deficiency Virus
WHO: World Health Organization.
